# Supplementary material for: DNA Methylation Influences miRNA Expression in Gonadotroph Pituitary Tumors
Source: Life (Basel). 2020 May 13;10(5):59. doi: 10.3390/life10050059 (PMC7281098; doi:10.3390/life10050059)

Supplementary Figure 1. Validation of selected small RNA molecules as the reference for normalization of qRT-PCR data for the assements of miRNA level in gondotroph pituitary neuroendocrine tumors

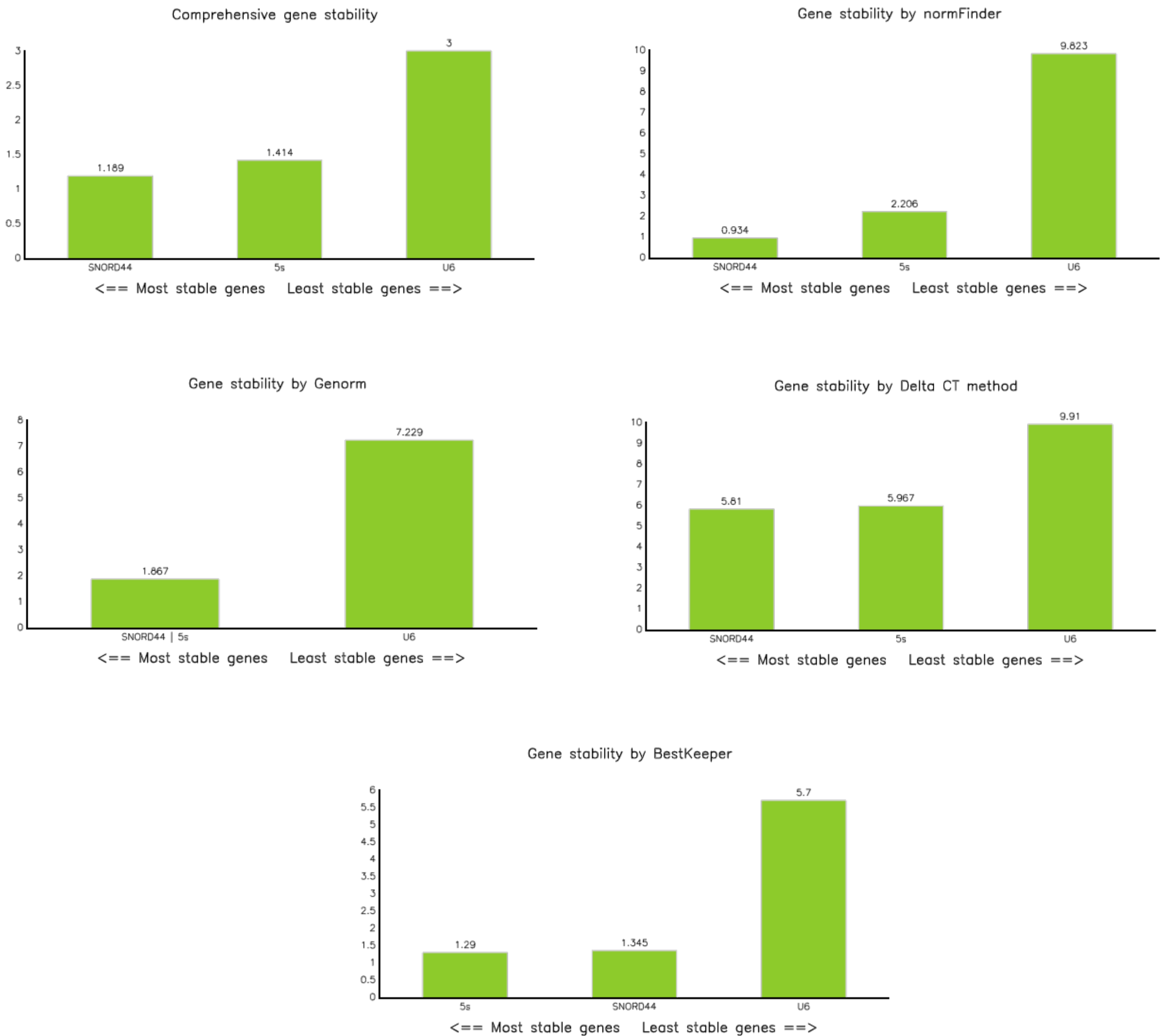

Supplement: Supplementary file 1 [file life-10-00059-s001.zip › life-775478-supplementary/life-775478-Supplementary Figure 1.pdf]
